# Thermal physiology and movements of skipjack tuna (*Katsuwonus pelamis*) from tag releases off the northern coast of Japan: Possible insights into spawning and wintering strategies

**DOI:** 10.1371/journal.pone.0336857

**Published:** 2025-12-02

**Authors:** Yuya Ueda, Yoshinori Aoki, Naoto Matsubara, Tetsuro Senda, Tomomi Tanaka, Fumiya Tanaka, Masachika Masujima, Yuichi Tsuda, Hidetada Kiyofuji

**Affiliations:** 1 Fisheries Stock Assessment Center, Fisheries Resources Institute, Japan Fisheries Research and Education Agency, Yokohama, Kanagawa, Japan; 2 Marino-Research Corporation, Kuwana-shi, Mie, Japan; Havforskningsinstituttet, NORWAY

## Abstract

Understanding movements for spawning of tunas is essential for gaining insight into population dynamics and can provide information on the reproductive characteristics of tuna stocks. Skipjack tuna spawn in tropical and subtropical areas and southward movement of presumed adult fish from the northern extents of their range may be related to spawning. We investigated southward movement patterns of skipjack tuna from the northern habitat limit of the northwestern Pacific Ocean using plastic dart tags and archival tag data. Observations uncovered a new alternative movement group that resides around the northern habitat at least for 9 months (residence group), confirming spawning potential movement toward tropical and subtropical areas (spawning potential group). The spawning potential group, that spent the majority of time in water temperature environments above 24°C in spawning grounds, was considered to be undertaking movement for spawning. Extraordinarily high body temperatures reaching 31°C were found only in the spawning potential group, indicative of spawning activity. The frequency and timing of this high body temperature were also consistent with the current reproductive traits of the skipjack tuna, strengthening this group as potential spawners. Conversely, gonadal immaturity of skipjack tuna in the residence group lasting above 8 months after release in environments unsuitable for spawning (<24°C) suggests motivation for movement was not driven by spawning, but by feeding and avoiding unfavorable environments. Remaining in the northern habitat with short-distance daily movement in a rich prey environment indicates engaging in feeding. However, seasonal cooling in this area pushed the temperature of the skipjack tuna thermal limit southward, leading to this group moving slowly southward to avoid exposure to the lower limit. The southward movement of this group was motivated by thermal limit avoidance. Physiological changes demonstrate the importance of describing the two movement patterns and underlying motivations for patterns from a physiological thermal tolerance and reproductive ecology perspective.

## Introduction

Long-term observation of the movement ecology of commercially exploited tuna stocks is crucial for evaluating their spatial and temporal shifts [1,2] and implications for the abundance and distribution of populations; hence, it is key for the sustainable use of their resources [3]. Migration in tuna is closely related to the spatial variability of resources and habitat suitability through their different life stages, such as feeding migration for juveniles [4,5], spawning migration for adults [6,7], and the movement of refugees from physiologically unfavorable environments through their life cycle [8]. From conservation and management perspectives, monitoring whether adult individuals alternate between spawning habitats, timing, and whether they are involved in spawning or skipping is particularly important, as these strongly affect population dynamics [6]. Despite their importance, direct connections between movement patterns and spawning behavior in tuna are predominantly observed in bluefin species, which exhibit distinct spawning locations confined to specific areas and seasons [9–12]. Monitoring the spawning migration of species with spawning habitats spread across wide areas throughout the year has proven challenging due to the inherent difficulty in reliably identifying the occurrence of spawning.

Physio-logging stands out as a promising tool for simultaneously tracking migration and its associated physiological changes in the body (e.g., Muhling et al. [4]; Fahlman et al. [13]; Aoki et al. [14]; Kitagawa et al. [15]). In particular, body temperature can be a useful indicator of physiological and ecological events of feeding and spawning, and could even be a warning sign of overheating [9,16–18]. Large thermal inertia and remarkably high heat production in tuna facilitate the maintenance of their body temperature well above that of the ambient environment [19,20]. However, this physiological advantage also poses the potential risk of overheating due to excessively high muscle temperatures, particularly during instances of burst swimming in warm areas [11,16,18]. The spawning behavior associated with active swimming in warm spawning grounds for bluefin tuna (*Thunnus orientalis*) is hypothesized to be a physiology–reproduction trade-off in the context of avoiding the risks of overheating [11]. This hypothesis, in turn, implies that observation of extraordinarily high body temperatures could be used to detect some specific behavior that does not occur during routine swimming activities, such as spawning behavior [10].

Skipjack tuna (*Katsuwonus pelamis*; SKJ) is a highly migratory species distributed in tropical and temperate areas [21,22]. Some of the individuals hatched in tropical and subtropical areas seasonally migrate toward the north and reach the northern limit of their distribution around areas off Japan in the Kuroshio-Oyashio transition area [21,23–25], which are known as rich prey areas for tuna [4,26,27]. Engaging in feeding in this area has the advantage of allowing the accumulation of energy for SKJ [26]. SKJ exhibits feeding migrations to this area and grows to approximately 50 cm fork length (FL) [25], which is sufficient to mature in the Pacific Ocean (the minimum length at maturity and length at 50% maturity (L50) are 40 cm and 50 cm, respectively [25,28]). Given that their spawning grounds are located in tropical and subtropical areas [28], adult SKJ at the northern limit move southward for spawning [21,29]. Though there is a rich body of literature focusing on northward movements [25,26], relatively few studies have investigated their southward movement, and it remains difficult to answer fundamental questions on southward movement, such as “Where do tuna migrate during southward movement?” or “What is the purpose of their movement?”. To gain a comprehensive understanding of SKJ movement ecology throughout their life history, it is necessary to fill this knowledge gap regarding southward movement, which is an ecologically important process for spawning.

The Japanese Tagging Program (JPTP) studies SKJ movement dynamics in the northwestern Pacific Ocean, dating back to 1966 and continuing through the present day. In 2011, the program implemented the use of archival tags (ATs), which record depth (m), internal (peritoneal) temperature, external (ambient) temperature, and light level (from which estimated positions are derived) to better define movements and behavior. The JPTP has focused on northward movement, targeting juvenile SKJ from the tropics and subtropics to the waters around Japan from January to July from 2010 to 2016 [25,26]. As a subsequent stage of this northward movement, focus has been placed on the southward movement of adult SKJ in the northern limit of their habitat, off Japan, from 2019 to 2022 (this study). The JPTP database comprises releases and recaptures information on dates, locations, and FL of tuna with PDTs for the entire study period, and ATs from 2011 to 2021. In addition to long-term data acquisition carried out by the JPTP and the advent of recent biologging techniques, the JPTP began collecting recaptured whole individuals after 2019, allowing further evaluation of their maturity stage based on direct histological observation of their gonads. In this study, we investigated the movement patterns of SKJ released in the northern extent of their range by classifying the individual movement patterns into two groups: 1) a spawning potential group that appears to seek a suitable habitat for spawning in tropical and subtropical areas, and 2) a residence group that remains in the offshore waters east of Japan. This study aimed to investigate the movement patterns of SKJ from their northern habitats. We examined the characteristics of each group, including the temperature environments experienced, thermal physiology (extraordinarily high body temperature), and reproductive traits. Finally, we discuss potential motivations for each group’s movement pattern, while checking for the possibility of involvement in spawning.

## Materials and methods

### Study area

**Fig 1:**
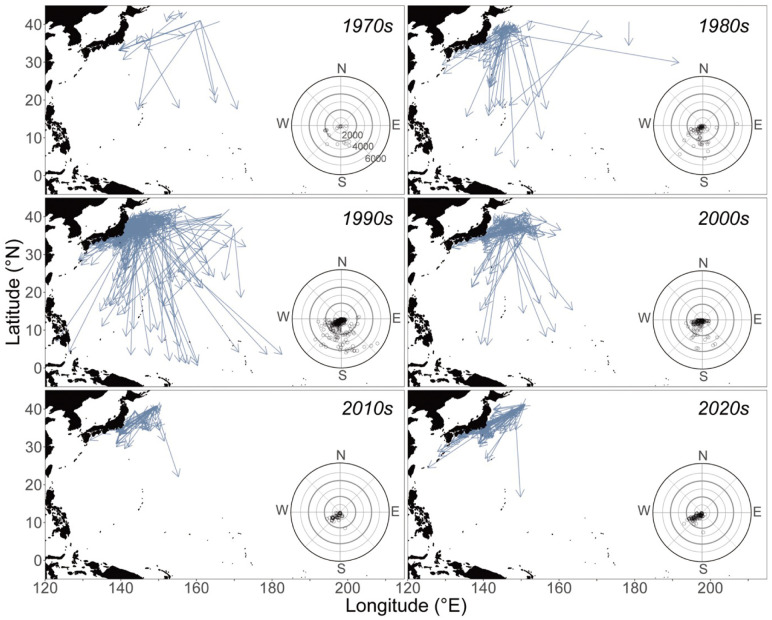
Decadal trends in the southward movement for SKJ tagged and released at the northern habitat limit (north of 35°). Arrows indicate the location of released and recaptured PDTs for the period from 1973 to 2022. Radar charts show the direction and distance (km) of movement between the release and recapture locations. Number of recaptured individuals in each decade is shown in Table 3.

This study was carried out in northern waters off-Japan. In the context of this study, the term ‘off Japan’ specifically refers to the Pacific side of the Japanese archipelago (**Fig 1**), and is consistently used to denote this region throughout the study. In this area, the warm Kuroshio Current and the cold Oyashio Current converge, resulting in a feeding ground for large predatory fish [4,26,27,30]. This temperature decrease intensifies as winter approaches, and by approximately October–November, the water temperature falls. Consequently, this is the northernmost extent of the northern movement of SKJ, which begin their southward movement after encountering this area.

### Tagging data

A comprehensive description of tag deployment has been previously published [25,26], hence, here, we briefly describe the tagging procedure. To investigate the southward movement of adult SKJ from their northern limit, tagging studies using ATs and PDTs were conducted between 2019 and 2022 in off-Japan (36° to 41°N and 141° to 149°E) as part of the JPTP, chartering a commercial vessel (No. 8 Nissho-Maru). Individuals were tagged onboard with plastic dart tags (PDTs) (Hallprint, Australia) at the base of the second dorsal fin and an AT in the peritoneal cavity (Lat 2910, Lotek, Canada). ATs were set to measure internal and external temperature, depth, and light intensity at 30 s intervals. A total of 6301 SKJ (fork length: 36.7 ± 18.8 cm) were released, and 383 individuals were recaptured.

In addition to the above data, we used historical tagging data obtained from 1966 to 2022 (using ATs from 2010 to 2022) in tropical and subtropical regions, off-Japan, and coastal areas along the Pacific side of Japan by chartering research vessels or commercial vessels. For these JPTP datasets, the following data filtering was applied to focus on individuals exhibiting southward movement and align with the range of the study area from 2019 to 2022: individuals released north of 35°N and whose recapture latitude moved at least 0.1° south of the release latitude for PDTs and individuals released in northern habitats from 2019 to 2021 were selected for ATs, in addition to two individuals that showed southward movement in a previous study on northward movement (ID: 2737, 2815 [25]). This filtering allowed us to analyze 856 recaptured individuals relative to the release point south with PDTs from 1973 to 2022, and 14 individuals tagged with ATs released in 2014, 2019, 2020, and 2021 (Tables 1 and 2). The FL (expressed as mean ± S.D.) of the individuals with PDTs was 48.8 ± 8.6 cm in the 1970s, 45.6 ± 4.7 cm in the 1980s, 47.0 ± 8.1 cm in the 1990s, 48.9 ± 9.2 cm in the 2000s, 43.4 ± 6.8 cm in the 2010s and 39.2 ± 9.1 cm in the 2020s. Of the 96 recovered ATs, 14 were analyzed in this study, as the rest stopped measuring early or recorded abnormal values for water temperature or light intensity that prevented accurate location estimation. Release and recapture information on the dates, latitude, longitude, FL, body weight, and days at liberty for the ATs used in this study are listed in **Table 2**.

**Table 1:** Information on individuals released with PDTs north of 35°, by decade.

Number of individuals				Number of recaptures relative to the release point		Days at liberty of recaptures relative to the release point: (Mean (range))	
Decades	Release	Recapture	Recapture rate (%)	North	South	North	South
1980s	1029	257	25.0	176	81	50.9 (1–338)	165.2 (0–1167)
1990s	12837	643	5.0	233	410	78.0 (1–698)	171.3 (2–1016)
2000s	8804	999	11.3	836	163	42.2 (0–389)	123.6 (4–882)
2010s	2350	180	7.7	118	62	35.4 (1–328)	221.1 (4–528)
2020s	7168	301	4.2	161	140	33.0 (0–374)	206.8 (4–599)

**Table 2:** Release and recapture information of ATs used in this analysis.

	Release				Recapture					
Tag No.	Date	Lat.	Long.	FL (cm)	Date	Lat.	Long.	FL (cm)	Weight (kg)	Days at Liberty
8943	Oct. 25, 2021	36.72	141.47	54	Apr. 28, 2022	33.54	136.52		5.1	185
8950	Oct. 25, 2021	36.43	141.47	53	May 19, 2022	30.83	138.67	60		206
9222	Nov. 4, 2021	36.85	141.32	54	May 23, 2022	32.48	133.20	60	4.6	200
9244	Nov. 3, 2021	36.70	141.33	54	Jun. 8, 2022	33.75	139.97	61	5	217
8947	Oct. 25, 2021	36.72	141.47	54	Jul. 4, 2022	33.50	136.08	61	5.25	252
9147	Oct. 25, 2021	36.43	141.47	56	Jul. 7, 2022	33.94	138.82	69.5	6.95	255
9149	Oct. 25, 2021	36.72	141.47	55	Jul. 14, 2022	34.52	139.27	62	5.5	262
8312	Oct. 16, 2020	40.98	150.9	35.5	May 11, 2021	32.33	136.83	45.5	2.1	208
6977	Oct. 3, 2019	39.17	143.98	50	May 7, 2020	34.30	137.30	64	5.1	218
6955	Oct. 7, 2019	39.52	143.70	53	May 16, 2020	33.50	139.77			223
6962	Oct. 7, 2019	39.52	143.70	49	Jun. 2, 2020	33.42	139.58	63.1	53	240
6949	Oct. 7, 2019	39.68	143.62	54	Jul. 7, 2020	36.00	147.00	69	6.9	275
2737	June 2, 2014	31.59	144.23	44	Sep. 9, 2014	27.37	129.43	50	2.2	100
2815	June 2, 2014	31.59	144.23	43	Mar. 30, 2016	22.58	149.42	70	6.4	668

### Geolocation estimates for the individuals with ATs

The daily locations of the individuals with ATs were estimated using light intensity records. We applied the Template fit method [31] available from the tag manufacturer’s software (LAT Viewer Studio) to estimate the latitude and longitude based on the local day length and the time of local noon. However, because the few differences in day length by latitude around the vernal and autumnal equinoxes make latitude estimates impossible, we modeled the light-based estimates using sea-surface temperature (SST) derived from external temperatures measured onboard the tags and those from remotely sensed satellite imagery within the unscented Kalman filter model in R software (UKFSST, https://github.com/positioning/kalmanfilter/wiki). The SST for the tagged individuals was averaged above a depth of 5 m, and satellite images were downloaded from NOAA’s 0.25-degree Daily Optimum Interpolation Sea Surface Temperature (OISST, https://psl.noaa.gov/data/gridded/data.noaa.OISST.v2.highres.html).

### Classification of two movement groups and their physiological traits

The thermal range for SKJ spawning is 24°C or higher [32]. In the western Pacific region, the areas that maintain such water temperatures year-round are located south of approximately 25°N ([28], S1 Fig). Accordingly, individuals that migrated into these areas were categorized as the spawning potential group, while those that remained north of 25°N were classified as the residence group. We first checked the number of individuals in each group by decade and then assessed the proportion of spawning potential groups among individuals with PDTs in each decade. To account for differences in sample size between years, we calculated the weighted mean and weighted standard deviation of these proportions, using the number of recaptures in each year as weights. This approach ensured that years with few recaptures did not disproportionately influence the decadal averages. Finally, we analyzed the water temperature experienced, thermal physiology, and reproductive traits to characterize the movement ecology of each group.

### Water temperature experienced

Daily average water temperatures experienced by individuals with ATs were derived from water temperature records, and monthly changes were examined for both migratory groups. The water temperatures experienced by each migratory group were compared with the physiologically important water temperatures for spawning activity (24°C [28]) and lower limit of lethal water temperature (18°C [33]). While SST was used in this study to infer spawning activity because of the availability of this data, it may not accurately reflect the actual thermal environment of skipjack tuna, which creates a slight mismatch between temperature experienced and SST. However, this mismatch is small enough to be ignorable, as spawning SST values (~24°C) correspond well with egg hatching temperatures [34] and larval distribution patterns [21,35]. Regarding the lower limit of water temperature, skipjack tuna may occasionally encounter very cold water for short periods [36]. However, to match the daily mean temperatures recorded by tags, here, we define the lower limit of lethal water temperature as the temperature that can be sustained throughout the day based on captive experiments [33]. Among the two spawning potential groups (Tag IDs 2737 and 2815), one individual (Tag ID. 2815) included a data period showing northward movement before moving south of 25°N. Therefore, only the data corresponding to the period when the individual started to move south of 25°N were included in the analysis.

### Thermal physiology

Thermal physiology can be a key indicator of tuna ecology, as high body temperature relative to water temperature may indicate specific physiological events in accordance with metabolic activity (e.g., Brill et al. [37]; Bernal et al. [20]). In addition, extraordinarily high body temperatures outside of the thermal limit for tuna could be a risk factor for overheating [18,33,38]. Here, we first checked the time series of peritoneal cavity temperature (hereafter, referred to as body temperature) and water temperatures for both groups and examined the relationship between body and water temperatures, with a particular focus on the daily maximum body temperature compared to the daily average water temperature to investigate the possibility of spawning comparing extraordinarily high body temperatures with experienced water temperatures as environmental index for spawning.

Subsequently, we classified the thermal physiology traits into two groups to confirm whether our classification of the two groups (spawning potential and residence groups) could be distinguished based solely on thermal traits. Pairs of variables (i.e., daily maximum body temperature and daily average water temperature, N = 2,702) were grouped using a hierarchical agglomerative clustering algorithm with a predetermined cluster number of two, assuming that thermal physiology can be classified into two groups: spawning potential and residence groups. The dissimilarity matrix was calculated based on Euclidean distances using the group average method available in the hclust function of the Stats package in R version 4.3.2 [39]. To focus on instances of extraordinarily high body temperature, we extracted the body temperatures greater than 31°C as the thermal limit, assuming the maximum upper body temperature limit for core red muscle of SKJ to be 35°C [33,38], and estimated the thermal difference between red muscle and the peritoneal cavity to be 4°C. The difference is based on the estimated thermal gradient within SKJ, where the red muscle is approximately 5°C warmer than the ambient water [40], and the peritoneal cavity is less than 1°C warmer than the ambient water under the experimental conditions [26]—indicating a temperature difference of about 4°C between the red muscle and the peritoneal cavity.

### Gonadal Index (GI) and ovarian histological observation

Direct observation of the maturity level of released and recaptured tagged individuals provides a more reliable method for inferring their movements. We examined the spawning potential of the residence group by examining the ovarian tissues of SKJ at the time of release and recapture during tagging in 2020 and 2021. As it is impossible to sample ovaries without dissection, we took ovaries from caught from the same school as the tagged individuals (N = 26, FL: 45–73.6 cm) as indices at release. Although we could only collect individuals with PDTs, we collected recaptured individuals as fresh samples (5–346 days at liberty). We measured the FL and gonad weight (Gw) of the individuals and fixed the gonads in formalin immediately after dissection. For ovarian histological observations, we collected the center of the formalin-fixed ovaries and embedded them in paraffin using an automatic paraffin-embedding device (Autokinet, Shirai Matsu Instrument Co., Ltd., Japan). Subsequently, we cut paraffin specimens into 6–8 μm thick tissue sections, which were double-stained with Mayer’s hematoxylin-eosin. We identified the most developed oocyte stages (perinucleolus, yolk follicle, yolk globule, germinal vesicle migration, hydrated oocytes, post-ovulatory follicle, α atretic oocyte, β atretic oocyte) and the degree of regression (IA) under a compound microscope to determine maturity using criteria in previously published literature for SKJ [28,41]. We also calculated the Gonadal Index (GI = Gw/FL^3^ × 10^4^, Gw: Gonad weight (g), FL: Fork length (cm)) in this study to evaluate more successive changes in maturity. Although the Gonadosomatic Index (GSI = Gw/Bw × 10^2^; Gw: Gonad weight (g), Bw: Body weight (g)) is generally used, we adopted GI to compare our results to those of a previous study in the same study area [28].

## Results

### Decadal trends in the southward movement of spawning potential and residence groups

Changes in southward movement from the 1970s to the 2020s indicated that the range of southward movement extended west of 180°E in the midwestern Pacific of the Northern Hemisphere (**Fig 1**). Overall, a high frequency of recapture was observed off Japan (north of 25°) (residence group), while some individuals also moved to southern tropical (0–15°N) and subtropical (15–25°N) areas, which encompass known spawning areas (spawning potential group). These trends were also observed in the 1970s; however, due to the small number of recaptures (45), the sample data from the 1970s were excluded from subsequent quantitative analyses.

The weighted proportion mean of spawning potential groups in the 1980s (**Table 3**) was the highest (22.22 ± 7.04%) and remained above 10% until the 1990s. However, this has decreased to below 10% since the 2000s. Focusing on the variation in each decade (**Table 3**, **Fig 1**), the variation from the 1980s to 2000s ranged from 5.83 to 7.04%, which was higher than that in the 2010–2020s (0.28–3.90%). Notably, the minimum latitude for spawning potential groups changed over the decades, approaching released areas (approximately 20°N) in the 2010s and 2020s, whereas they reached the equator in the 1980s, 1990s, and 2000s.

**Table 3:** Decadal frequency of spawning potential group. The weighted mean is calculated from the percentages of spawning potential groups within each decade.

Decades	Number of individuals		Weighted proportion mean ± SD of spawning potential groups per year (%)	Minimum latitude of recapture (°N)
	Spawning potential group	Residence group		
1980s	18	63	22.22(±7.04)	2.18
1990s	53	359	12.86(±7.30)	0.60
2000s	15	152	8.98(±5.83)	5.97
2010s	1	61	1.61(±0.28)	22.08
2020s	2	138	1.43(±3.90)	16.73

### Comparison of the recaptured position with position estimates from individuals with ATs

**Fig 2:**
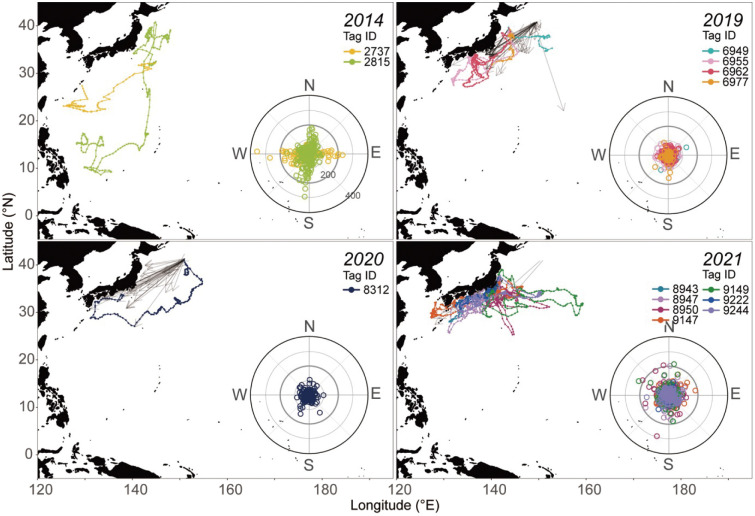
Daily position estimates obtained from ATs of SKJ released in the period from 2014 to 2021. Colored lines represent the daily position estimates of each individual. Radar charts show the daily direction of movement and the distance (km/day). Release and recapture locations of PDTs shown as arrows are overlaid for comparison.

**Fig 3:**
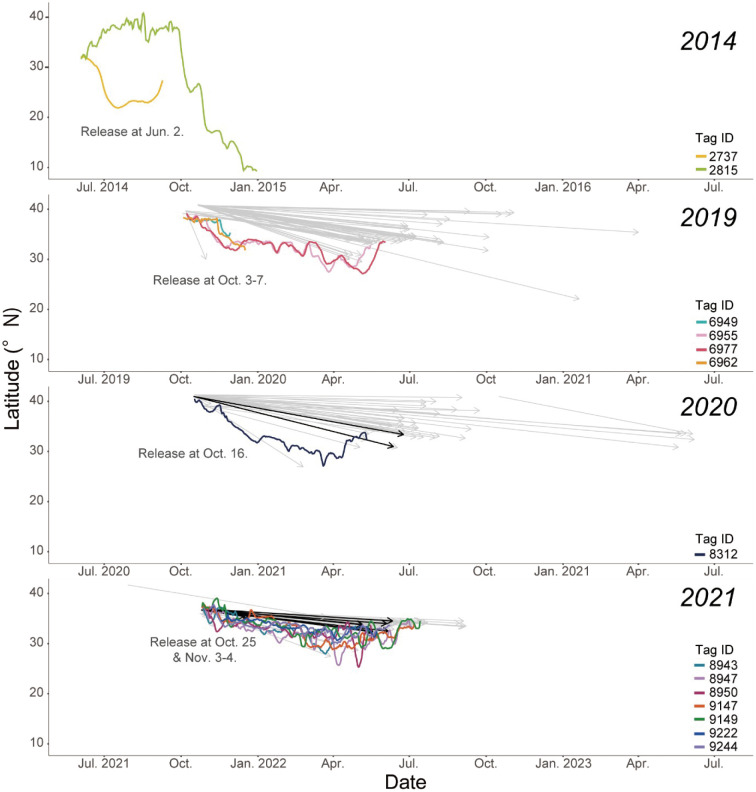
Comparisons of latitudinal movements between ATs and PDTs of SKJ released in periods from 2014 to 2021. Colored lines represent the change in latitude of the estimated location of each individual with the AT. Arrows indicate the latitude of the recapture position of the released PDTs. Black arrows indicate individuals with female gonad samples.

The estimated movement of the ATs indicated that the two individuals released in 2014 were classified into the spawning potential and 12 individuals released from 2019 to 2021 were classified into residence groups, respectively (**Figs 2 and 3**). Two individuals from the spawning potential group (2737 and 2815) were released in June. Tag ID. 2737 began to move southward following release and reached approximately 20°N after 100 days at liberty. Tag ID. 2815 moved northward after release before turning southward in October, reaching approximately 10°S after 668 days at liberty. In contrast, 12 individuals from the residence group were released from October to November and began to migrate southward in November-December, reaching latitudes of 28–30°N (**Fig 3 and** **S1 Fig**).

A comparison of the position estimates from ATs with known recapture positions of individuals with PDTs released from the same school showed that the recaptures of individuals with PDTs were concentrated around the movement paths estimated from ATs (**Figs 2 and 3**). Although all individuals with ATs were recaptured by July of the following year (**Fig 3**), some individuals with PDTs were recaptured around 28°N in April-May after moving southward from the release area, and others were recaptured at 40°N in October, the same location as the release area (**Fig 3**). The quantitative movement characteristics of both groups were examined using the distance and direction moved per day. We found that individuals in the spawning potential group had temporary periods of strong preference in their direction with large movements (average distance in October at 2815:76.8 ± 61.1 km/ day, in June at 2737:82.0 ± 58.3), whereas all individuals in the residence group tended to move shorter average distances of 34.6 ± 27.6 km/day with no particular directionality (**Fig 2**).

### Difference in water temperature experienced between the two groups

**Fig 4:**
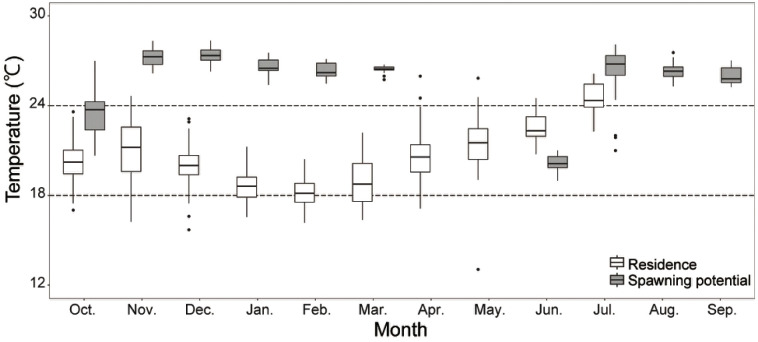
Monthly changes in average daily average water temperature experienced for the residence and spawning potential groups. Dotted lines at 18°C and 24°C represent the lower thermal limits of SKJ habitat [33] and potential spawning habitat [28], respectively. The residence group (N = 12) was released in October 2019–2021 and the spawning potential group (N = 2) was released in June 2014. Note that the data for spawning potential group in the period from June to September only includes the Tag ID. 2737 as the other individual (Tag ID. 2815) moved northward (>30°N) in this period (See **Fig 3**).

Comparison of water temperatures experienced by the spawning potential and the residence groups (**Fig 4**) showed that the water temperature experienced by the spawning potential group (25.9 ± 1.9°C) was higher than that of the residence group (20.0 ± 1.8°C). It is noteworthy that the water temperatures experienced by the spawning potential group were above 24°C throughout the year, which is the temperature threshold for spawning, except for the months when they were in temperate areas in October and June (**Fig 3**). The water temperatures experienced by the residence group were 18°C from January to March, which is considered to be the lower thermal limit. On a daily scale, the minimum temperature was 15.7°C, which was recorded by one individual (Tag ID. 9222), and the minimum record for the monthly average temperature was 18.2°C in February. Most individuals experienced temperatures above the lower limit of the habitat temperature. A comparison of the distribution of tagged individuals and environmental water temperatures around Japan in winter also showed that SKJ were distributed near the 18°C isotherm and that SKJ moved southward in synchrony with the southward movement of the 18°C isotherm (**S1 Fig**). The water temperature experienced by the residence group increased from 20.5 ± 1.4°C to 24.4 ± 1.1°C in the months from April to July, indicating that even for this group, the water temperature experienced was conducive for spawning in July.

### Characteristics of thermal physiology in two groups

**Fig 5:**
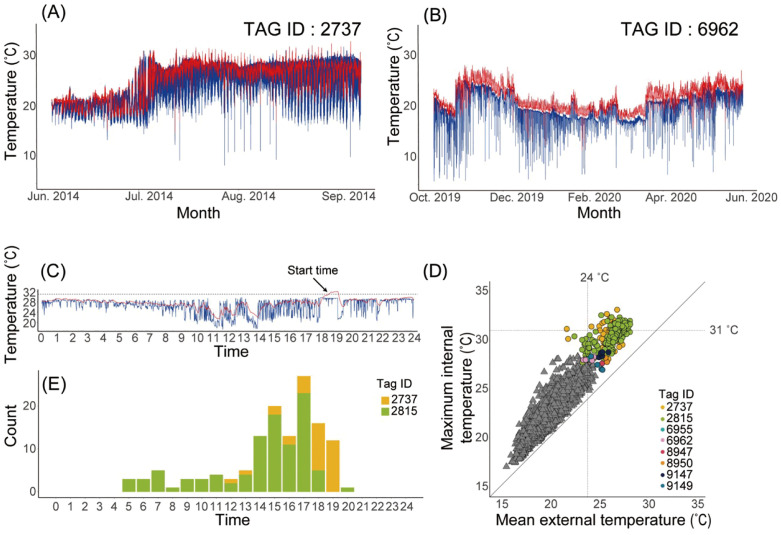
Examples of body temperature and water temperature experienced records during southward movement (A and B) and the features of the extraordinarily high body temperature (C-E). Time-series of body temperature (red line) and water temperature experienced (blue line) for the spawning potential (A, Tag ID. 2737) and residence (B, Tag ID. 6962) groups. Example of body and water temperature experienced within a single day (day, month, year) during which abnormal temperature increases occur for the individual (Tag ID. 2737) **(C)**, relationship between maximum daily body temperature and average water temperature experienced **(D)**, and frequency of the start time when abnormal temperature increases occurred **(E)**. **(C)**: Criterion for an abnormal temperature increase is 31°C (cited), and the arrow indicates the time of its onset. **(D)**: Hierarchical clustering has classified the data into circles and triangles. The colors of the circles indicate the respective individuals. The horizontal dotted line indicates 31°C, an indicator of extraordinarily high body temperature, and the vertical dotted line indicates 24°C, the lower limit of the temperature at which spawning is possible (cited). **(E)**: Horizontal axis represents the onset time of the abnormal temperature increase, coinciding with the arrowed onset time in **(C)**. Each bar represents the number of instances of abnormal temperature increase for each individual.

Examples of the time series of body temperatures and water temperature experienced during days at liberty for spawning potential (Tag ID. 2737) and residence groups (Tag ID. 6962) are shown in **Fig 5A** and **Fig 5B**, respectively. The thermal difference between the body and water environments in the spawning potential group (Tag ID. 2737) showed only a small difference over the entire period from June to September (**Fig 5A**). In contrast, the thermal difference in the residence group (Tag ID. 6962) was more profound from October to April but tended to decrease after May (**Fig 5B**).

The individual in the spawning potential group (Tag ID. 2737 and TagID. 2815) showed an extraordinarily high body temperature; for instance, for TAG ID. 2737, the temperature started increasing at approximately 18:15 HRS, reaching 32°C over the next 35 minutes, lasting for 15 minutes (Fig 5A and 5C). Subsequently, the body temperature dropped after a rapid decrease in water temperature (Fig 5C). These were observed at intervals of up to three days between July 12 and 26 and August 12 and September 4.

To investigate the possibility of spawning during the detection of extraordinarily high body temperature based on water temperature experienced data, the relationship between the daily maximum body temperature and daily average water temperature was explored for all individuals (**Fig 5D**). The maximum body temperature increased slightly with the warm water temperature. Clustering analysis based on this relationship distinguished the two distinct groups. This grouping is roughly explained by warm and cool water clusters. Both individuals in the spawning potential group (Tag IDs 2737 and 2815) and another six individuals from the residence group aggregated in the warm cluster. From these data, we extracted body temperatures exceeding 31°C as extraordinarily high body temperatures, which resulted in only two individuals from the spawning potential group. Time of occurrence of an extraordinarily high body temperature was between 5:00 HRS and 20:00 HRS (**Fig 5E**), and the frequency was higher in the period between 14:00 HRS and 18:00 HRS (**Fig 5E**).

### Reproductive traits in the residence group

**Fig 6:**
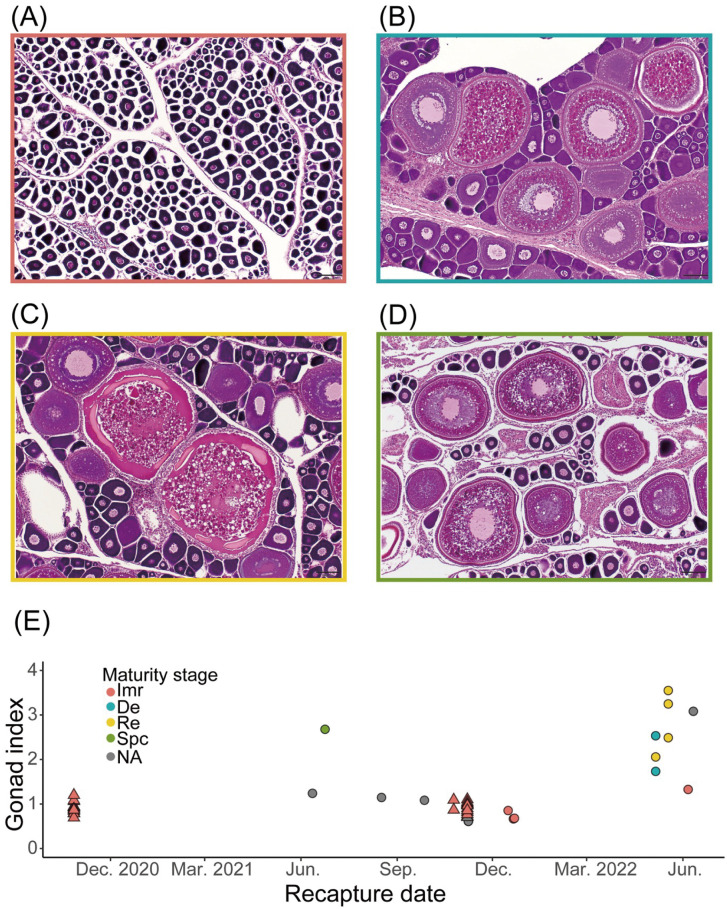
Monthly changes in gonadal index (GI) and maturity stage of recaptured female SKJ. Sampled individuals (N = 19) available for maturity analysis were released in 2020 and 2021. **(A-D)** Photographs of the oocyte. Maturity stages were determined based on ovarian histological observation, with colors representing stages: immature (Imr; **A)**, developmental (De; **B)**, regressive (Re; **C)**, and spawning capable (Spc; **D)**. **(E)** Triangles represent individuals sampled at release, indicating the released group, and circles represent recaptured individuals. Colors denote maturity stages, while gray indicates individuals unavailable for observation.

Successful recovery of the whole body for individuals recaptured off Japan in 2020 and 2021 (N = 19), as well as alternative samples from the same school with released individuals onboard, enabled us to investigate GI with maturity level estimates (Table 4, **Fig 6**). Overall, all individuals with a GI below 1.33 were immature, and those with a GI above 1.33 showed multiple maturity stages. Maturity level at release for both 2020 and 2021 is estimated to be immature with a small standard deviation (N = 26, GI = 0.94 ± 0.13). Level of development did not change after release, but variation in the stages, such as development (N = 2, GI = 1.73 and 2.53) and regressive (N = 4, GI = 2.84 ± 0.88) phases, began to appear after May. Only one individual recaptured in June 2021 showed spawning capability (GI = 2.68) in June, which was almost eight months after release.

**Table 4:** Histological characteristics of the ovarian maturity phase and gonadal index (GI) and gonadosomatic index (GSI) of female SKJ from residence group.

Sampling at	Maturity stage	n	MAGO	POF	IA (%)	Mean GI ± SD (Range)	Mean GSI ± SD (Range)
Release	Immature	25	Pn or Ca	Absent	Absent	0.94 ± 0.13(0.71-1.21)	0.41 ± 0.06(0.25-0.51)
Recapture	Immature	4	Pn or Ca	Absent	Absent	0.88 ± 0.31(0.67-1.33)	0.44 ± 0.18(0.33-0.72)
Recapture	Developmental	2	Py or Sy or Ty	Absent	< 50	2.13 ± 0.57(1.73-2.53)	0.98 ± 0.23(0.82-1.15)
Recapture	Regression	4	Py or Sy	Absent	≧50	2.84 ± 0.69(2.06-3.55)	1.31 ± 0.34(0.96-1.72)
Recapture	Spawning capable	1	Sy	Present	<50	2.68 ± -(2.68-2.68)	1.24 ± -(1.24-1.24)

Pn: perinucleolus oocytes; Ca: cortical alveolar; Py: primary yolked oocytes; Sy: secondary yolked oocytes; Ty: tertiary yolked oocytes; POF: postovulatory follicles; MAGO: most advanced group of oocytes; n, number of specimens; IA: relative intensity of atresia.

## Discussion

Southward movement of SKJ from the northern extent of their range is hypothesized to be a spawning movement toward the main spawning areas of tropical and subtropical areas [21,29]; however, there is a lack of evidence-based data to test this presumption. This is the first study to identify southward movement patterns with direct measurements of movement using substantial historical tagging data over a period of almost half a century. Observations based on rich data uncovered a new alternative residence group residing around the northern habitat and confirmed the previously presumed spawning potential group. We described the two movement patterns (spawning potential and residence groups), as well as the potential motivation for each pattern from the perspective of physiological thermal tolerance and reproductive ecology by applying the physio-logging methodologies for measuring changes in thermal physiology and histological observation of the gonad, along with movement tracking. We first evaluated whether the spawning potential group was actually involved in spawning, and discussed the characteristics of the newly discovered residence group, focusing on why they did not move to the main spawning areas of the tropical and subtropical regions. Finally, we noted the limitations and assumptions of this study.

### Implication for involvement in spawning in the spawning potential group

The spawning potential group spent most of their time in water temperatures above 24°C in spawning grounds and was considered to be undertaking spawning movement. The first indication that this group is a spawning potential group can be inferred from the conditions that meet the reproductive ecology of the wild. The temperature experienced in this group (>24°C) and body size (>43 cm) meet the minimum standard required for spawning (24°C and 40 cm [28]) in known spawning areas of the tropics and subtropics; however, compared with the L50 (50 cm), the individual (TAG ID: 2737) at the time when spawning behavior was first detected was slightly smaller, although they had reached a size of 50 cm at recapture in September. Additionally, behavioral characteristics showing strong directionality and long-distance movements southward from the northern habitat to the spawning areas would imply a strong preference to search for more preferable environments by leaving temperate areas. The structural path in marine animals plays an important role in navigating goal areas [42]. The straight movement observed in the spawning potential group suggests that it is possible for SKJ to orient spawning areas by exploring warmer areas.

In addition to circumstantial evidence and behavioral implications, extraordinarily high body temperatures in the spawning potential group in warm tropical and subtropical areas would also indicate their involvement in spawning [9–11]. Possible factors for this extraordinarily high peritoneal cavity temperature are unusual physiological events, including heat increment during feeding [5,17,26]. However, extraordinarily high body temperatures were observed only in the spawning potential group, under temperature conditions conducive to spawning (>24°C), which would not be explained by feeding ecology but is specific to behavior in spawning [10]. The fact that this phenomenon was observed at an interval of 1–3 days is consistent with the spawning frequency of SKJ, which spawn at intervals of 1–4 days [28,32]. Furthermore, spawning behavior occurred from 14:00 HRS –18:00 HRS, which was again in broad agreement with the time of appearance of the final mature phase (15:00 HRS –21:00 HRS [43]). Courting and spawning behaviors in bluefin tuna have been observed along with vibrating caudal fins [10]. Such burst-swimming behavior is likely to generate heat [9,11,12]. The sex of the recovered individuals for this group is unknown, but behavioral activity during spawning would be active in both males and females. Although further validation of the elevation in body temperature is necessary using captive SKJ with visual confirmation under controlled experimental conditions [44], as well as to obtain supporting evidence through the histological analysis of ovaries from tagged individuals that exhibited extraordinarily high body temperatures in the wild, the consistency of these high body temperatures with reproductive ecology would suggest that this elevation in body temperature is associated with spawning behavior.

The overheating risk involved in active swimming in warm spawning grounds is hedged by behavioral thermoregulation [11]. We provided the first validation of the physiological hypothesis in wild SKJ that the unique thermal physiology in tuna causes overheating [11,16,18]. We found the peritoneal cavity temperature to have reached a maximum of 33°C. Although no actual threshold for overheating temperature in the peritoneal cavity is available, some studies hypothesize the overheating temperature for SKJ core red muscle to be 35°C [33,38] and the 4°C difference in temperature between peritoneal and core red muscle would suggest that the peritoneal temperature over 31°C result in potential overheating [18,38]. Despite facing this risk, a rapid drop in body temperature, (after elevation) associated with dive behavior serves to cool down the body by releasing body heat into the deep, cold environment [11]. This remarkable behavior could be the behavioral thermoregulation observed in tuna [45,46], which would be necessary for SKJ to undertake while avoiding overheating in warm areas. In addition, the low thermal differences above the ambient temperature in the spawning potential group may also imply thermoregulatory ability by physiologically changing thermal conductance in warm areas [9,11,15].

### What made the residence group stay in the northern habitat with slight southward movements?

First, we evaluated the possibility of spawning in the residence group to better understand the motivation behind their behavior. Although gonadal samples were limited to a small number of individuals tagged with PDTs, water temperature data recorded by ATs provided valuable insights into the environmental conditions potentially inferring spawning activity in this group. Unlike the spawning potential group, the average water temperatures experienced by the residence group remained below the spawning threshold of 24°C ([28]) until July, suggesting that this group occupied a temperature range unsuitable for spawning during this period. Consistent with this observation, gonadal index (GI) values one month after release were low (0.94 ± 0.53, [28]), indicative of an immature phase. GI values in this study showed a slight increase (1.73–3.55) from May to June 2022 but remained lower than those observed in actively spawning individuals (5.13 ± 2.38, [28]). Histological observations also revealed no spawning-capable individuals during this period. However, one spawning-capable individual was observed in June 2021. While results from this single individual are associated with high uncertainty, they align with previous findings that spawning occurs north of 25°N in June [28]. These findings support the hypothesis that spawning in the residence group begins in summer, which is about 9 months after release. This suggests that spawning is not the primary driver for their behavior. Further research involving a larger sample size is necessary to substantiate these conclusions.

The motivation for their residence would be explained by feeding and avoiding unfavorable environmental conditions. The areas where the residence group was distributed after release correspond to the Kuroshio-Oyashio transition zone, known for its rich prey environments [4,26,27,30]. Individuals distributed near the northern limit of SKJ habitats off Japan feed in prey-rich areas [26]. Additionally, the observed movement patterns in the residence group involved small, random movements without significant direction. This behavior aligns well with the characteristics of the Levy flight foraging theory in prey-abundant environments [47], which suggests that individuals remain in areas with sufficient prey and move significantly only when prey availability declines. Given that the timing of leaving temperate areas differed between residence and spawning potential groups (**Fig 3**), differences in residency and migratory behavior between the two groups may be linked to seasonal changes in food prey. However, even for individuals that remained resident, seasonal cooling in this area pushed the 18°C thermal limit [33] for SKJ further south, suggesting that the residence group would need to gradually move southward to avoid exposure to lower temperatures. Therefore, the movement pattern in this group appears to be driven by a combination of feeding in prey-rich environments and avoiding the lower thermal limit due to seasonal cooling.

### Limitations and assumptions of this study

We classified each group by applying a previously defined distinct boundary (25°N) in the reproductive ecology of the northwestern Pacific Ocean [28]. Classifications based on geographical features may be arbitrary. However, an additional clustering analysis based solely on thermal physiology in relation to water temperature showed the same classification results for individuals (Tag ID. 2737 and 2815) classified into the spawning potential group. This result is reasonable, as behavior and related thermal physiology are specific to spawning grounds [9–12]. Thus, the same grouping results based on thermal physiology and geographical features would strengthen the validity of using two groups based on geographical features.

PDTs used in this study collect large amounts of data, but the quality of the PDTs is biased owing to the spatial variability in fishing effort, reporting rate, and tag mixing [48,49]. However, the quantity of data from ATs is small, primarily owing to the high cost of the device [50,51], but their data are independent of fishery locations and seasons, making data obtained from ATs of a higher quality than those from PDTs [51]. Well-covered recapture locations for the PDTs within the distribution range of the ATs from 2019–2021 would suggest that the shortfall in data quality for PDTs and that of quantity for the AT could be compensated for by each other. This comparison is not extendable to all data periods due to the lack of AT data for the historical period; however, the consistency of the locations between tags allows us to assume that the recapture locations identified with the PDTs are sufficient to discuss SKJ ecology.

The decrease in the residence group and the proportion of the spawning potential group over recent years suggests potential changes in movement patterns. However, the tagging data depends on the recapture process through fisheries, which limits the ability to draw conclusions regarding movement changes. Since the recapture of PDTs depends on fishing activity, the recapture rates in areas with limited fishery operations are likely to be underestimated. Specifically, the fishing grounds for Japanese pole-and-line operations in tropical and subtropical waters have contracted over time [52]. This contraction may lead to an underestimation of recaptures from the spawning potential group, particularly individuals located south of 25°N. To examine changes in movement patterns over time, future studies will need to correct for these biases by incorporating catch and effort data for each fishing area. Additionally, an individual-based modeling approach that considers fishing mortality is a key direction for future research [53].

## Supporting information

S1 Fig Daily distributions of tagged SKJ with sea surface temperature (SST) in the period from October 2022 to July 2023. Each colored circle represents an individual, and the numbers in the legend indicate the tag ID. The two dotted isotherm lines show the physiologically important temperatures: a minimum spawning-capable temperature of 24°C [28] and lower thermal limit of 18°C [33]. We used the SST developed by the Japan Fisheries Research and Education Agency based on the Regional Ocean Modeling System (FRA-ROMSII) with three-dimensional variational analysis schemes [54].
(DOCX)

S1 File Spreadsheet containing underlying data. Column names are defined on the first sheet. (XLSX)
