# Supplementary figures and images for: Thermal physiology and movements of skipjack tuna (Katsuwonus pelamis) from tag releases off the northern coast of Japan: Possible insights into spawning and wintering strategies
Source: PLoS One. 2025 Dec 2;20(12):e0336857. doi: 10.1371/journal.pone.0336857 (PMC12671813; doi:10.1371/journal.pone.0336857)

**Supporting Information Fig S1**


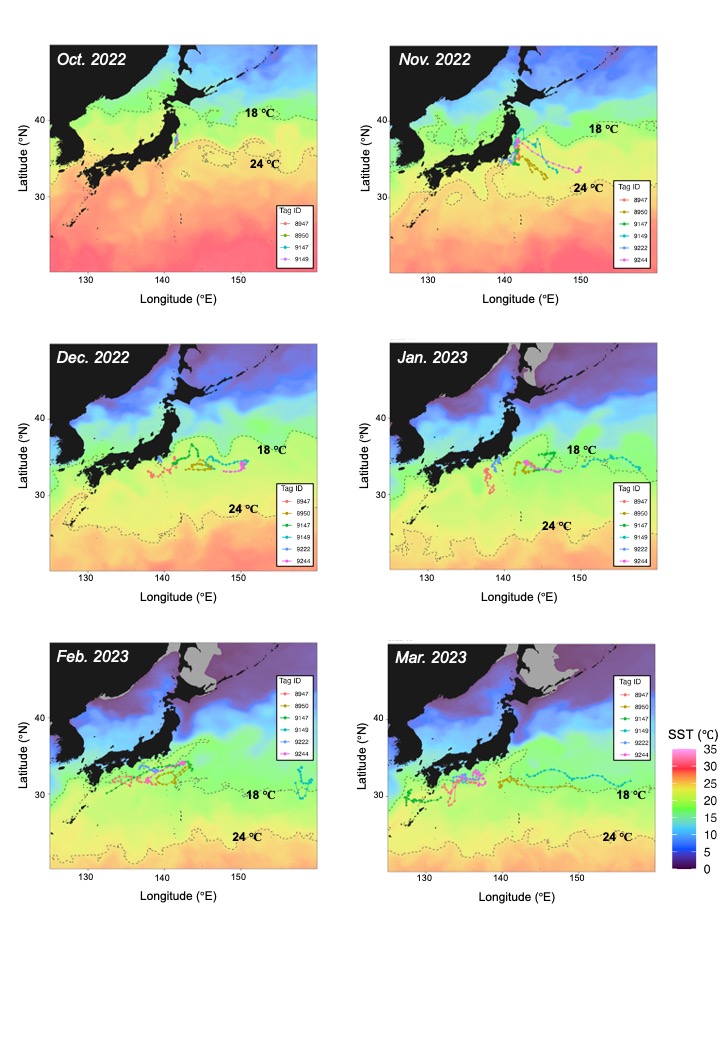


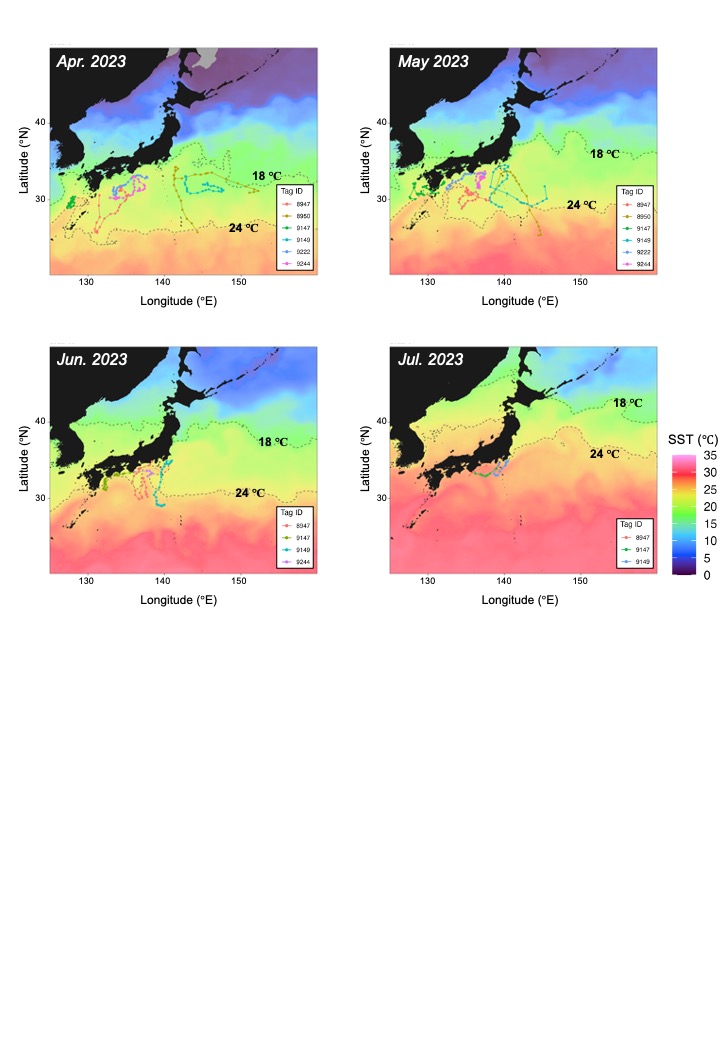

Supplement: S1 Fig — Each colored circle represents an individual, and the numbers in the legend indicate the tag ID. The two dotted isotherm lines show the physiologically important temperatures: a minimum spawning-capable temperature of 24°C [28] and lower thermal limit of 18°C [33]. We used the SST developed by the Japan Fisheries Research and Education Agency based on the Regional Ocean Modeling System (FRA-ROMSII) with three-dimensional variational analysis schemes [54]. (DOCX) [file pone.0336857.s001.docx]
